# Mediastinal Paraganglioma: A Challenging Surgical Approach

**DOI:** 10.7759/cureus.72198

**Published:** 2024-10-23

**Authors:** Lucas N Canaan, Deepak Vivekanandan, Allen Barbarovich, Karen Gersch

**Affiliations:** 1 Surgery, Northeast Georgia Medical Center Gainsville, Gainesville, USA; 2 Surgery, Trinity School of Medicine, Warner Robins, USA; 3 Cardiothoracic Surgery, Northeast Georgia Medical Center Gainsville, Gainesville, USA

**Keywords:** mediastinal paraganglioma, mediastinal tumor, paraganglioma, robotic surgery, thoracic surgery

## Abstract

Mediastinal paragangliomas, though rare, present significant surgical challenges due to their proximity to critical vascular structures within the mediastinum. This case report discusses the management of a patient with an incidentally discovered non-functional mediastinal paraganglioma. The tumor's location necessitated meticulous preoperative planning and intraoperative navigation to prevent vascular injury. Successful excision was achieved through a multidisciplinary approach involving precise imaging and intraoperative techniques to manage the distorted anatomy. Post-operative outcomes were favorable, with no vascular complications or recurrence observed during routine follow-up. This case underscores the importance of careful surgical planning in managing mediastinal paragangliomas in a multidisciplinary fashion and the importance of a high degree of clinical suspicion in the absence of classical symptoms.

## Introduction

Mediastinal paragangliomas present a rare and complex challenge because of their unique characteristics and the specific location within the mediastinum. Born of neural crest-derived chromaffin cells situated in the para-aortic ganglia, these tumors constitute a distinctive entity within the spectrum of sympathetic nervous system neoplasms. Unlike their more prevalent adrenal counterparts, mediastinal paragangliomas manifest outside the adrenal glands, often along the sympathetic chain within the mediastinum [1]. 

The clinical manifestation of mediastinal paragangliomas is nonspecific and oftentimes incidentally diagnosed, with up to 50% of patients displaying an absence of symptoms. This frequently leads to discovery through various imaging modalities or post-mortem examinations. When symptomatic, manifestations usually arise bimodally due to either excessive catecholamine secretion, compression of neighboring anatomical structures, or both. Symptoms may include hypertension, palpitations, diaphoresis, cephalalgia, and anxiety, reflective of catecholamine excess, or encompass features associated with compression of adjacent organs, such as dysphagia, dysphonia, cough, thoracic discomfort, or, in rare instances, superior vena cava syndrome precipitated by venous obstruction secondary to tumor enlargement [1, 2]. 

Diagnosis of these masses, especially when asymptomatic, typically requires a multifaceted workup integrating imaging modalities such as computed tomography (CT) scans, magnetic resonance imaging (MRI), and functional studies like metaiodobenzylguanidine (MIBG) scintigraphy or positron emission tomography (PET) scans. Histopathological scrutiny following biopsy or surgical resection corroborates the diagnosis, often revealing characteristic histological features, including nests or clusters of cells known as “zellballen,” surrounded by sustentacular cells and separated by segmenting bands of fibrovascular stroma [3,4]. 

Given their propensity for aggressive behavior and potential for metastasis, early diagnosis and intervention are paramount. However, the management of mediastinal paragangliomas poses formidable challenges due to the intricate anatomical relationships they maintain with critical structures such as major blood vessels, the heart proper, and bronchopulmonary parenchyma. While complete surgical resection remains the cornerstone of therapeutic intervention, achieving maximal tumor extirpation while minimizing collateral tissue damage requires meticulous surgical planning, often requiring a multidisciplinary approach involving cardiothoracic surgeons, vascular surgeons, and neurosurgeons [4]. 

Despite advancements in surgical techniques and perioperative care, the prognosis for mediastinal paragangliomas remains guarded, particularly in cases of advanced disease or incomplete resection. Vigilant long-term surveillance is imperative to monitor tumor recurrence or metastasis, mandating periodic imaging assessments and biochemical evaluations to detect residual or elucidate recurrent disease [4-6].

In recent years, targeted therapeutic modalities such as molecularly guided treatments and peptide receptor radionuclide therapy (PRRT) have emerged as promising avenues in the management of advanced or metastatic mediastinal paragangliomas, furnishing additional therapeutic options for patients who may not be amenable to surgical intervention or who experience disease progression despite surgical excision [7]. Nonetheless, further investigation is warranted to refine treatment algorithms and ameliorate outcomes for patients grappling with this rare yet potentially life-threatening condition, and the paucity of literature on these masses suggests that there is great utility to the analysis. 

## Case presentation

**Figure 1:**
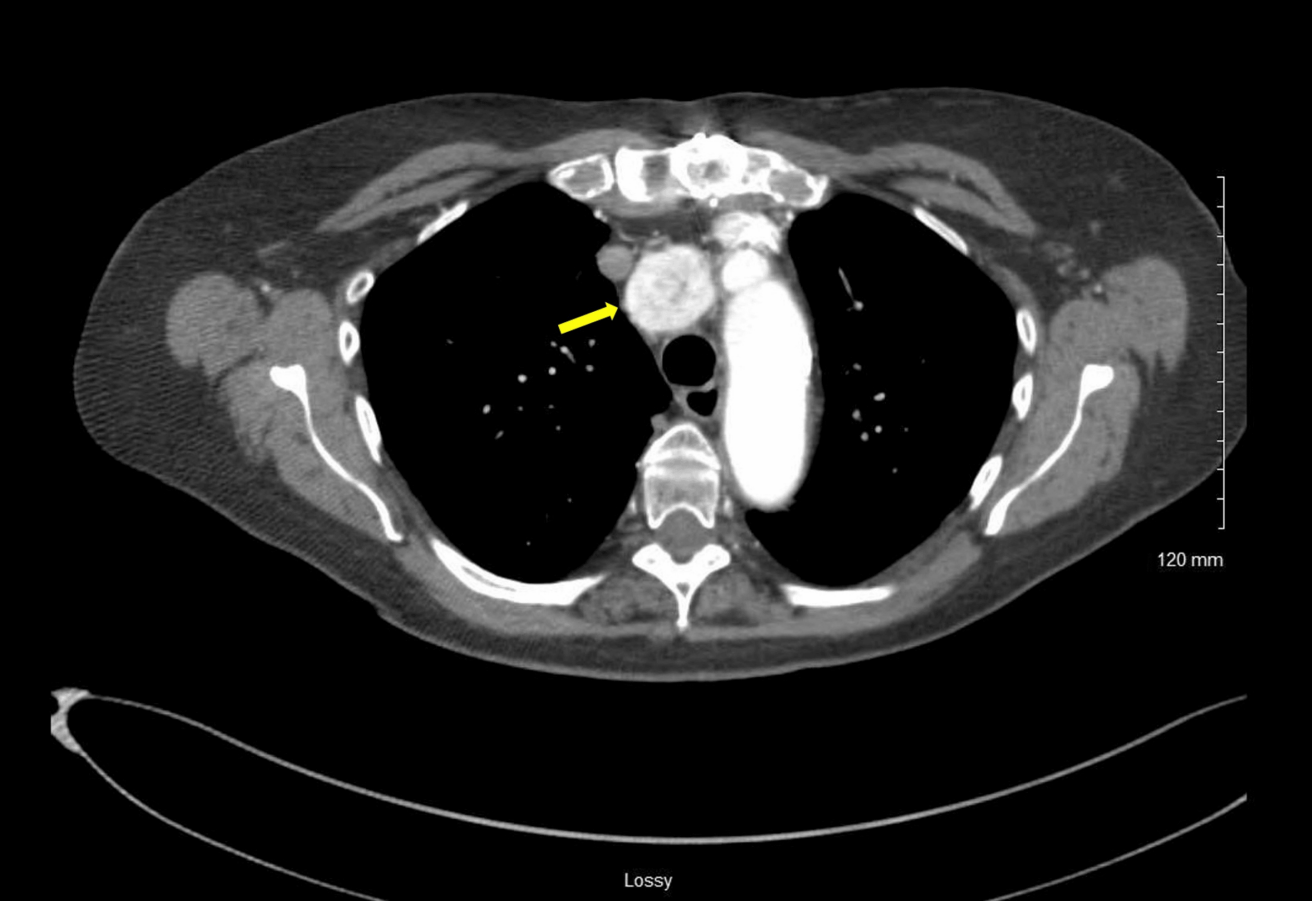
Axial cuts of a preoperative computed tomography of the chest with IV contrast demonstrating a right paratracheal enhancing mass (yellow arrow)

**Figure 2:**
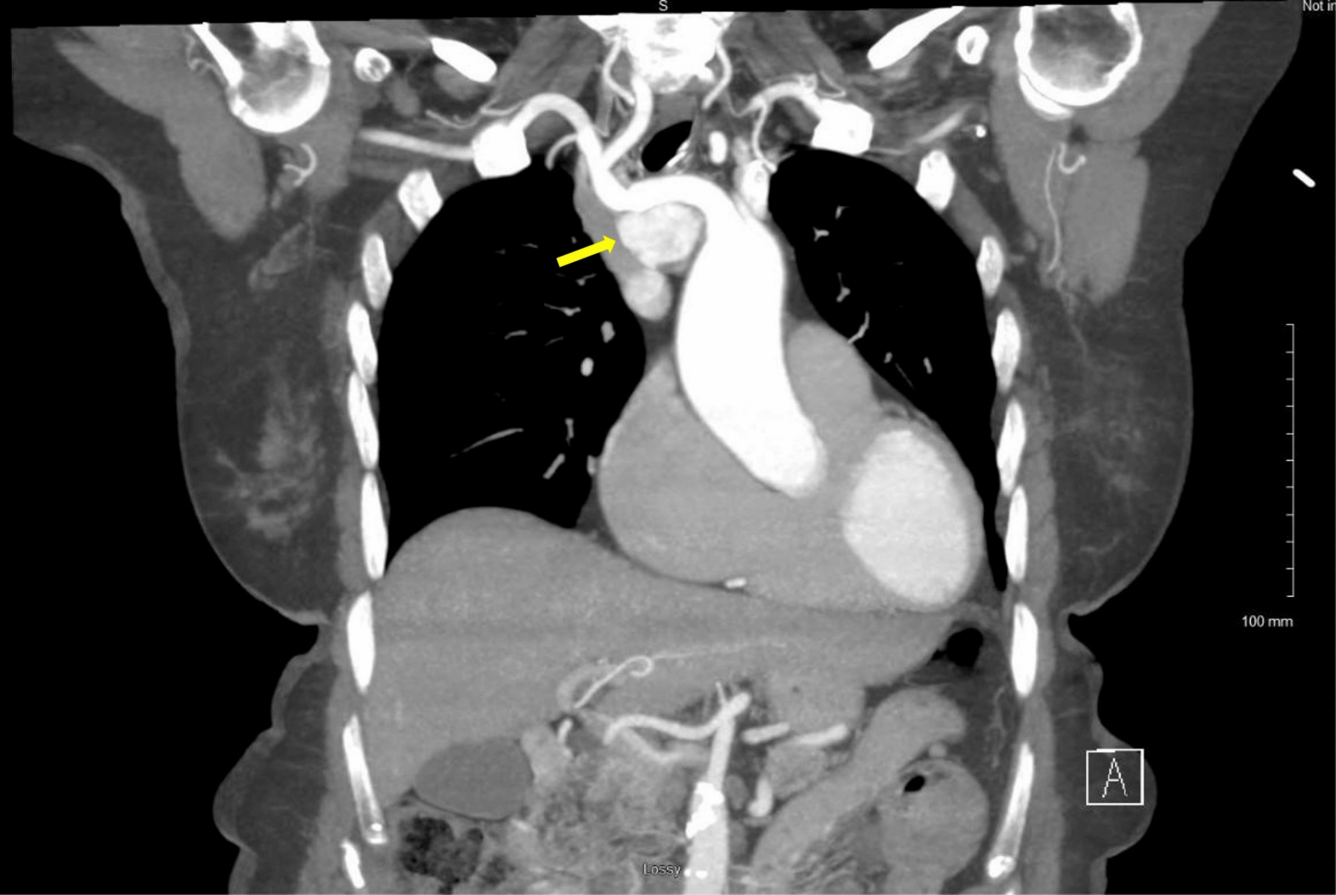
Coronal view of a preoperative computed tomography of the chest with IV contrast demonstrating a right paratracheal enhancing mass (yellow arrow) and its intimate proximity to the great vessels of the mediastinum.

An 82-year-old female patient with a history of mitral valve replacement presented to the clinic after the incidental discovery of a mediastinal mass during a transthoracic echocardiogram (TTE) for surveillance of her replaced mitral valve. In this patient's case, the mitral valve was replaced using an open surgical approach, which involved a median sternotomy to access the heart. After placing the patient on cardiopulmonary bypass, the mitral valve was accessed through the left atrium. The damaged valve was removed and replaced with a bioprosthetic valve, after which the left atrium and sternum were closed. As with many surgical procedures, operating in areas with previous dissection presents unique challenges due to adhesions and scarring. On initial evaluation, the patient denied any symptoms associated with mass during her interview. Furthermore, she denied any symptoms such as headache, palpitations, or diaphoresis, which would be consistent with a catecholamine-secreting tumor. She presented the clinic with a chest CT with IV contrast, which showed an enhancing mass in the right paratracheal region of the mediastinum measuring 28 mm x 34 mm (Figures 1, 2). The CT did not show any other concerning findings. The imaging did help delineate the involvement of the great vessels of the mediastinum. Based on this imaging, it would not be possible to biopsy the specimen via interventional radiology. It was decided at that time the patient would need surgical resection of the mass. 

The surgical approach selected for this patient was robot-assisted right-sided video-assisted thoracoscopic surgery (VATS) using the DaVinci Xi system (Intuitive Surgical, Inc., Sunnyvale, CA). This minimally invasive approach was selected due to the optimal window being present from the right side based on pre-operative imaging. This approach was also beneficial as the patient's replaced mitral valve was done through a sternotomy, which significantly increased the risk of scarring and a very difficult dissection if the mass resection was done in an open fashion. 

Upon entering the chest, the mass was clearly identified just anterior to the trachea and posterior to the superior vena cava that extends towards the aorta and the origin of the innominate artery. Dissection began at the posterior aspect of the mass, followed by the anterior and inferior margins. Upon dissecting the superior surface of the mass, an area was encountered that offered no appropriate plane to continue with the dissection. The margin of the mass abutted the innominate artery, making dissection treacherous. Efforts to identify planes for dissection were hindered by the distorted anatomy caused by both the mass and previous surgical procedures. Alternate planes were explored, following the path of the previous surgical access, but no safe option was identified. At this stage, due to the proximity of the mass to critical structures and the inability to identify a safe plane of dissection despite exhausting all available options, the decision was made to take biopsies of the mass rather than proceed with total excision for the safety of the patient. After the biopsy samples were harvested and meticulous hemostasis was achieved, the ports were removed, and a single 24-French Blake chest tube was placed. 

Her post-operative course was largely uneventful, and she was discharged from the hospital post-operative day one after the removal of her chest tube. She was scheduled to follow up in the clinic for further discussion of the next steps following the results of the pathology. 

Outcome and follow-up 

Pathology 

A sample of the mediastinal mass and two suspicious lymph nodes were taken during the surgery and sent for pathology. These were taken from stations 7 and 10R. The mediastinal mass was determined to be a paraganglioma but stained positive for chromogranin, synaptophysin, and CD56 immunostains. The Ki-67 was approximately 1% to 2%. The tumor was negative for PAX-8, TTF-1, melan-A, CDX-2, cytokeratin 7, cytokeratin 20, pancytokeratin, and GATA-3. Neither lymph node showed definitive atypia or malignancy.

Post-operative Follow-Up

**Figure 3:**
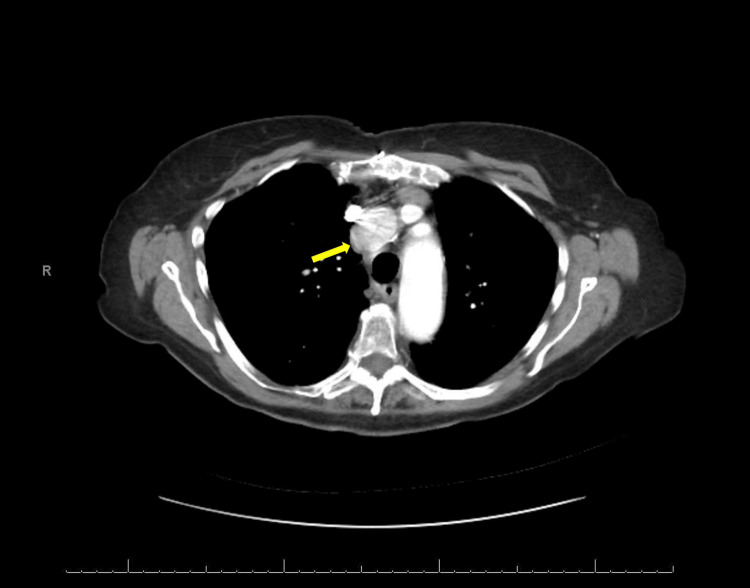
Axial cuts of a post-operative CT scan of the chest with IV contrast demonstrating a stable mass in the mediastinum (yellow arrow)

The patient was seen in the clinic two weeks after surgery and was doing well. Her incisional pain had largely resolved. She denies any post-operative shortness of breath and continues to deny any symptoms related to the mass. A discussion was held with the patient about a referral to oncology for ongoing management. Given the complex and adherent nature of the tumor, it was recommended to forgo further surgical resection and instead proceed with observation from a surgical standpoint. The patient was seen by an oncologist who recommended external beam radiation therapy (EBRT) for further management. The patient was agreeable to this plan of care, and she was referred to radiation oncology. A review of her medical record from the radiation oncologist was that she would receive 50 Gray (Gy) in 25 fractions. The patient completed her radiation therapy on May 7, 2024, and tolerated it well. The most recent CT chest with IV contrast shows the mass is stable from previous observations with no additional concerning findings (Figure 3).

## Discussion

The diagnosis and treatment of this 82-year-old female patient reflect not only the rarity of mediastinal paragangliomas but also the complex nature of management when one is discovered. Oftentimes, in the presence of functional paragangliomas, the presenting symptoms lead the clinician down the appropriate diagnostic path. When presented with a nonfunctioning paraganglioma, the prominent symptom will be compression of surrounding structures [8, 9]. This provides a unique challenge in management. In this patient’s case, it was discovered incidentally on surveillance of her mitral valve. Not all patients are so fortunate. On imaging, the mass showed high enhancement features [8]. Another confounding factor within this patient’s case and not completely uncommon to other patients is the ability to biopsy the mass. The mass is routinely around critical structures of the thorax, which can make windows for biopsy difficult [8]. This patient did not have appropriate windows to safely biopsy the mass with the assistance of interventional radiology. 

Her therapy progressed in a manner consistent with other case reports and common practices from the sources reviewed [8-10]. The more unique aspect of her care revolves around surgical management. As minimally invasive surgery continues to grow and become a mainstay in both abdominal and thoracic surgery, the additional capabilities provided by robotic assistance are particularly noteworthy in this case. Dissection around the great vessels of the mediastinum would have been tremendously difficult with traditional VATS, and in this patient’s case, given her previous sternotomy, open surgery would have also posed significant risks. The significant advantage offered by robotic assistance in this case is the improved visualization provided by the camera, as well as the enhanced articulation of the robotic arms. These factors greatly improved the ability to delicately dissect around the mass. Despite these advantages, the mass could not be removed due to its adherence to the great vessels of the mediastinum. 

Overall, the key takeaways from this case are the rarity of the tumor and the surgical approach. Mediastinal paraganglioma was somewhat unexpected in pathology. After diagnosis, the patient proceeded through surgery followed by radiation therapy. Surgical resection was attempted, followed by adjuvant radiation dosed to 5,000 centigrays (cGy) divided into 25 treatments, which she tolerated well. Significant consideration was given to this surgery. The use of minimally invasive techniques with robotic assistance provided the best surgical outcome and an optimal post-operative course. The patient experienced minimal pain after surgery, had only a few small incisions, and spent a short time in the hospital. By leveraging modern techniques and technologies, the best outcome was achieved, allowing the patient to recover and continue her life. 

## Conclusions

This case report highlights the diagnosis and management of mediastinal paraganglioma. It emphasizes the complex nature of the surgical approach and the difficulty of the initial diagnosis. Many patients are asymptomatic, only finding the mass incidentally on other imaging modalities and during unrelated investigations. A typical workup for both pheochromocytomas and paragangliomas starts with the evaluation of metanephrines and normetanephrines, staging imaging, and genetic counseling. Further workup and imaging can be indicated if there is concern for multifocal or metastatic disease. After biochemical workup and diagnosis of paraganglioma, surgical resection is the mainstay of treatment, with the primary goal of complete resection for the mediastinal paraganglioma. The surgical approach can often be difficult due to the mass being adherent to the critical vascular structures of the mediastinum, making dissection more difficult. In this case, this was further confounded by the patient having a prior mitral valve replacement, making an open approach less desirable due to extensive post-surgical scarring. 

Depending on surgical resectability, treatment can continue in two different directions. If the mass can be completely resected, surveillance is done via chemical testing and imaging. For unresectable diseases, as in this patient’s case, treatment can vary based on the recommendations of medical oncology, ranging from surveillance to radiotherapy and adjuvant systemic chemotherapy. In this patient’s case, she chose to proceed with radiation therapy. This case highlights the importance of a high index of clinical suspicion in the workup of all pathologies for unrelated and incidental findings. Patients seldom present with a focal disease, and it is the responsibility of the entire medical team to activate the appropriate diagnostic consultations and ameliorate holistic medical treatment for all conditions that contribute to the patients’ health. These incidental findings often become quite complex both from a surgical and medical treatment standpoint, and the cooperation of a multidisciplinary team is vital to ensuring the best outcome for patients. 
